# Do COVID-19 and Food Insecurity Influence Existing Inequalities between Women and Men in Africa?

**DOI:** 10.3390/ijerph19042065

**Published:** 2022-02-12

**Authors:** Heather VanVolkenburg, Isabelle Vandeplas, Katim Touré, Safiétou Sanfo, Fatoumata Lamarana Baldé, Liette Vasseur

**Affiliations:** 1 Equal Contribution—Department of Biological Sciences, Brock University, 1812 Sir Isaac Brock Way, St. Catharines, ON L2S 3A1, Canada; hvanvolkenburg@brocku.ca; 2 Center for International Studies and Cooperation (CECI), 3000 Omer-Lavallee, Montreal, QC H1Y 3R8, Canada; isabellev@ceci.ca (I.V.); fatoumata.balde@ceci.ca (F.L.B.); 3 Département Economie et Sociologie Rurales, École Nationale Supérieure d’Agriculture (ENSA), Université Iba Der Thiam de Thiès, Thies BP A 296, Senegal; katim.toure@univ-thies.sn; 4 Laboratoire de Développement Agricole et Transformation de l’Agriculture (DATA), Université Thomas Sankara, Ouagadougou 03 BP 7210, Burkina Faso; sanfo.safy@gmail.com

**Keywords:** West Africa, Sub-Saharan Africa, marginalization, at-risk, gender-based violence, gender inequalities, food insecurity

## Abstract

This review sought to understand what is currently known about how the ongoing COVID-19 pandemic and restrictive measures are affecting food security and equality between women and men in all of Africa. A review of both the academic and grey literature was performed by following PRISMA guidelines. Results showed that a general disparity exists in gender-inclusive/-sensitive research. Most reported increases in inequalities between women and men were predictive only. Evidence-based articles found were mainly conducted online and target tertiary educated populations, among which neutral effects were found. A general lack of disaggregated data (e.g., women vs. men) was found to be a barrier in gaining a complete understanding of the situation on-the-ground. Furthermore, documents reporting on food security seldom included all four pillars (i.e., availability, access, utility, stability) in their analysis despite the reciprocal connection between them all. Within household disparities and the impacts on power relationships within households were also overlooked. Future studies must focus on rural settings and gender disaggregated interview processes as well as consider all pillars of food security. Doing so will help to better inform governments and humanitarian groups leading to better designed policies and social supports that target where they are most needed.

## 1. Introduction

Food insecurity is a reality experienced globally, but this is especially true in many countries of Africa. The State of Food Security and Nutrition in the World (SOFI) report from FAO, IFAD, UNICEF, WFP, and the World Health Organization (WHO) states that in 2020 over 2.3 million of Africa’s people were suffering from acute food insecurity [1]. Eighty percent of food production in Africa relies on smallholder farmers who have little access to and/or cannot afford advanced farming technologies [2]. Many factors can exacerbate the vulnerability of people to food insecurity. These include but are not limited to violent insurgencies and counterinsurgencies leading to displacement and livelihood disruptions, limited state presence, and competition over access and use of agricultural land and natural resources [3]. Extremes in climate variability due to climate change and degraded resources attributed to overuse and abuse of the land are also stressors that make sufficient production difficult to achieve [3,4].

Since March 2020, the world has been dealing with an additional stressor, the COVID-19 pandemic, which currently pushes the world economy into a recession. The progress made in recent years regarding the UN SDGs has either halted or regressed worldwide [5,6]. In Africa, the COVID-19 pandemic and subsequent restraint measures are driving countries toward an economic and social crisis where not only health care but all spheres of life are affected, including food security. Most African countries rely heavily on local commercial and subsistence agriculture production for food security. Despite this, even before the pandemic, many regions and countries of Africa were food insecure. The SOFI report identifies an increase of 3% in the number of hungry people in Africa; in a period of only one year, from 2019 to 2020 (21% of the population), the increase was the sharpest in Western Africa [1].

In Africa, women play a critical role in agriculture, representing two thirds of farm labourers; although Palacios-Lopez et al. argued that this number was more like 40%, this representation only included crop production [7,8]. Women are generally responsible—partly or entirely—for food production, distribution, and utilization [9,10]. The restrictive measures put in place to reduce the risk of transmission of the COVID-19 virus have increased the food insecurity of many individuals [11]. Given that women have such a prominent role to play in agriculture and food security, it is possible (likely even) that they have been disproportionately affected by the pandemic in terms of food security. For example, and as documented in the latest report on the State of Food Security and Nutrition in the World 2021, the gender gap in the prevalence of moderate or severe food insecurity has increased worldwide during the COVID-19 pandemic, as women were 10% more likely to be moderately or severely food insecure than men in 2020 as compared to 6% in 2019 [1]. It has been reported that, even prior to the pandemic, women faced higher levels of food insecurity than men [1]. Women often are burdened with responsibilities linked to unpaid work (e.g., providing care), or in food system-related roles [12,13].

In addition to food security, it has been predicted that other gender disparities, such as gender-based violence, would increase due to pandemic stressors [14,15]. However, whether speaking about food security or gender-based violence, or both, such reports lack clear evidence to support that those increases have happened or are lockdown-driven, and instead, tend to be more predictive and based on past crises (e.g., the Ebola epidemic; [16,17]) and media reports; see Saalim et al. for a comprehensive list of examples [18,19]. While the assessment of past crises and current media are valuable ways to help predict and mitigate gender inequalities driven by the current evolving crisis, neither provide concrete evidence that there is a link.

Here, we examined how the countries’ restrictive measures against the pandemic affected or exacerbated inequalities between women and men. The objective of this rapid review was to examine the various aspects of gender inequality when people are confronted with food insecurity related to the COVID-19 pandemic in Africa.

While articles are emerging on the impact of the COVID-19 pandemic on the economy and health, few report evidence related specifically to how power relations between women and men influence food security, or the reverse. Furthermore, few articles address food security and how it affects other issues that in turn affect women (e.g., pregnancy, mental-health, and well-being). We wanted to extract as much information as possible about the issues related to gender disparity linked to food security in this context using both scientific literature (using a PRISMA protocol) and grey literature (government and humanitarian group reports) to better understand the current view of the situation and especially identify the knowledge gaps.

This review constitutes the knowledge base for an IDRC-funded research project implemented by the Centre d’études et de cooperation internationale (CECI) based in Canada, in partnership with the Centre d’Études, de Documentation et de recherche Économique et Sociale (CEDRES) in Burkina Faso, the École Nationale Supérieur d’Agriculture (ENSA) de Thiés in Senegal, and Brock University in Canada: “COVID-19, food security, and opportunities for reconfiguring unequal gender relations in Burkina Faso and Senegal”. This research project assesses the impact of the pandemic and the measures implemented on food security and on gender relationships within communities within the pillars of food security (availability, access, utilization, and stability) in rural Burkina Faso and Senegal. Therefore, we expected that results from this review contribute to this research project to inform policy makers and humanitarian providers about how pandemics and various types of restrictions might affect food security to draw lessons for future pandemics.

## 2. Materials and Methods

While this rapid review was initially planned to address West Africa alone, only nine out of 28 articles were found that reported associations between COVID-19, food security, and gender inclusively (only five of which were not based on predictions of what may happen rather than evidence of what has happened). We therefore expanded the review to consider all of Africa. The initial step of this rapid review was conducted using PRISMA protocols, as explained in Vasseur et al. [11]. Briefly, PRISMA is an internationally recognized set of guidelines that are followed to create robust, repeatable review methodologies (see Tricco et al., 2018 for further details [20]). The original protocol from Vasseur et al. [11] is also registered on the Open Science Framework (https://doi.org/10.17605/OSF.10/PKH98, accessed on 13 January 2022). The first search was conducted in March 2021, while an updated search was performed on October 2021 at Brock University (Niagara Region, ON, Canada; ISP address 70.54.102.247). In addition to these searches, a new search was conducted by adding the search terms “AND (gender OR wom* OR female* OR girl*) to the previous search string. Articles that did not focus on COVID-19, gender and food security inclusively were discarded.

Any article that focused on or mentioned any pre-determined gender-related terms (i.e., “gender”, “wom”, “female”, “girl”, “marginal”, “vulnerable”) was retained for further screening (phrases were detected by using a search feature (in Adobe^®^ Acrobat^®^ software on the entire article). Relevant ‘hits’ were then checked for context/relevance of any of the terms that were present in the respective document. If any additional relevant references were discovered during the assessment of returned articles, they were also retained and screened as secondary findings. Once all articles were screened and relevant documents pooled together, each were subjected to an in-depth reading and extraction process.

Each article retained for final extraction was categorized according to the location of reference (either a country or region), which of the four pillars of food security that the article touched upon (availability, access, utilization, or stability), the type of article that was being considered (e.g., grey or peer-reviewed, qualitative and/or quantitative), and whether it was an original study, opinion, report or review. Media outlet documents were not considered for this review. Each article was further evaluated for whether or not it provided evidence or only predictions concerning the effect of COVID-19 and food security on existing inequalities between women and men in Africa (i.e., ‘category’). Finally, for those articles that were considered to provide evidence, a qualitative rating was applied to describe the overall message according to the evidence presented (this process was also applied separately to predictive documents). Ratings indicated whether inequalities between women and men remained neutral, were negative (i.e., women experienced less inequality than normal), positive (i.e., women experienced higher inequality than normal), or mixed (i.e., both women and men experienced context-dependent inequalities, sometimes higher, sometimes lower, sometimes both depending on the parameter) for women relative to men, or unknown based on insufficient evidence.

## 3. Results and Discussion

Of the 4583 screened documents, 1005 (22%) underwent a full text review with a further 76 (8%) subjected to the Adobe^®^ gender search term locator strategy. By the end, 28 of the 76 articles (37%) were retained for information extraction, three of which were used strictly as background material (see S1 for PRISMA flow chart). Of the 25 retained articles, 10 reported some evidence of how COVID-19 and food security was influencing preexisting inequalities between women and men in Africa (Ghana = 1, Kenya = 2 including 1 that also focuses on Uganda, Nigeria = 4, South Africa = 2 and West Africa, including Ghana, Nigeria, Niger, Liberia, Sierra Leone, and Guinea = 1). Seven of the evidence-based articles were peer reviewed. All other articles (*n* = 15) reported predictive information based on what may happen due to previous pandemics, gender disparities, or anecdotal information (Table 1). Therefore, on the initial 1005 articles that went for a full review, only 25 (i.e., 2%) had the combination of COVID-19 pandemic x food security x gender/women issues.

### 3.1. Pillars

Regarding food security, four out of the ten evidence-based articles focused on all four pillars (i.e., availability, access, utilization, and stability), albeit not always leading to the potential for interpretation of contextual error. The least referred to pillars were those of utilization (*n* = 4) and stability (*n* = 5) while access was referred to in no less than nine articles (availability *n* = 6). Of the retained predictive documents, more than half referred to all four pillars together (*n* = 8/15), and each individual pillar was found to be represented in over 50% of the retained articles. A more detailed discussion regarding the effects of the COVID-19 pandemic on food security pillars is presented in Vasseur et al. [11]. Predictions were clearly different than evidence-based research methods, and we must do a better job of designing research that focuses on all four pillars (preferably at the same time, as they are all connected).

### 3.2. Article Ratings

#### 3.2.1. Neutral and Mixed

Four of the 10 evidence-based articles analyzed in this study were interpreted as reporting neutral gender inequalities linked to COVID-19 and food security. Three were interview-based studies targeting mainly respondents with post-secondary education, which may have been due to the use of internet-driven surveys. Since an internet platform is often not accessible to all individuals in a population, these studies should be acknowledged as being somewhat unrepresentative of countries in general. Folayan et al. reported mixed results when interviewing 4439 Nigerians (53% of whom were women and 80.9% of whom had tertiary education) [25]. Overall, women and men reported similar financial losses (when no other variables were considered—with men reporting slightly higher at 58% compared to 55% for women). Food security was similar between men and women, with only 1% more women reporting being food insecure than men (21% vs. 20%). Overall, the odds of males being more food insecure were found to be lower than females; however, the authors admitted that gender was reported but not investigated to its fullest extent. Additionally, respondents who reported a negative impact of the pandemic on their daily life were mainly those with a primary school education. A separate gender analysis within this sub-sample was not conducted.

A study conducted by the Bayero University Kano’s Centre for Dryland Agriculture in Nigeria found that 75% of the women interviewed reported that no discrimination was placed on them by farm operators when attending markets [24]. However, these statistics must be interpreted carefully, as only 7/26 of those interviewed were women. The authors go on to suggest that this may have been due to women having higher workloads (e.g., care economy) due to restrictions and the inability to attend markets. It is also worth noting that individuals interviewed were mainly university staff, employed, and living in an urban setting, which does not necessarily give an accurate representation of the situation, as surveys would not have represented more vulnerable populations such as rural subsistence farmers, women-headed households or individuals that are not highly educated. Iddi et al. reported that 12% of Ghanaians surveyed through an online questionnaire about stress levels linked to well-being felt stressed about the possibility of getting COVID-19, thus causing food insecurity [21]. The study group was mainly composed of young people (56% under 34, 86% under 44, and 44% having no children), 79% lived in urban Ghana, 98.7% possessed a post-secondary education level, and 72% were employed by the government. Males and females’ feelings in this group were found to be quite similar. A more inclusive interview process (e.g., all ages, all occupations, inclusive of rural environments) would present a clearer picture as to the current situation. Furthermore, HSRC echoed a similar sentiment regarding the lack of disaggregated data being a problem in South African studies [28]. Thus, disaggregation of any data that reports on women and men is an urgent need for future research.

A few studies looked at food security at the household level by performing a gender analysis using the head of the household. Although better than using data that are not disaggregated, looking at the gender issue at the level of head of household does not necessarily present a complete understanding of the situation that women face. For example, Ibukun and Adebayo conducted interviews to evaluate the state of food security at the household level during the COVID-19 pandemic in Nigeria [26]. Gender was not specifically analyzed, and results indicated that, education level income, and wealth status determined whether households reported being food insecure—of which more than half reported being severely food insecure. Moreover, the authors report that 10% of households in the North are female headed as opposed to 25% of the households in the South. The difference is significant and demonstrates that regional discrepancies may occur, and research should be conducted and reported accordingly so as not over-generalize. Furthermore, such studies completely omit the male and female relationships and how decisions are made within a household. This concern underlines the need for on the ground analysis of the conditions and the importance of interviewing women and men separately, as conditions can differ greatly.

Chitiga et al. present an interesting model that demonstrates differences in loss of income between female-headed and male-headed households (i.e., women losing more) using micromodel simulations based on gender differences in employment sectors to assess redistributive impacts of economic scenarios on poverty [31]. Their simulations indicated a higher impact on female poverty (increase by 5% from 46% to 51% in the severe scenario). Some of the model inputs relied on 2012 statistical data (e.g., poverty rates), and it wasn’t clear whether they accounted for any sort of government COVID-19 and food security assistance that could have been applied. It must also be understood that the model itself is still just predictive and not necessarily a reflection of what is happening. In addition, the simulation looks at the impact on household poverty and does not provide direct information about food security within households.

The remaining two articles in the neutral category focused on a more heterogeneous population. Chiwona-Karltun et al. found no differences between men and women’s likelihood of increased worries; however, they noted that 70% of respondents were from urban areas, supporting a need to understand whether differences would be found in rural settings [16]. Their conclusions were based mainly on predictions from past crises and evidence of gender disparity in other contexts (some from the 1990s). The complexity of highlighting gendered differences due to the social-construct of the concept of “food-worry” should be noted. Maula recommended that more work needed to be done towards collecting, reporting, and disseminating better data surrounding gender impacts, as the data were poor [15]. It is therefore difficult to confirm that gender inequalities continue to happen at the alarming rate with what is currently being reported in the literature, (even though many would agree that it is) or if COVID-19 is aggravating current situations.

#### 3.2.2. Higher Effect Reporting

Only two evidence articles were found to report higher inequalities faced by women linked to COVID-19 pandemic and food security. Liverpool-Tasie et al. conducted surveys focused mainly on hidden components of food supply chains and how they were negatively affected during initial pandemic stages [26]. While only two points were given concerning impacts to women, they were related to loss of employment and therefore a critical component of food security (e.g., Nigerian fish businesses went from supporting 20% to 2% female employment between February—pre-pandemic and April; however, males were also impacted, but to a lesser extent, with total man workers dropping to 20% from 50% during same period). In South Africa, interviews (*n* = 40; women = 22, men = 18) provided insight into women vs. men farmers [29]. While in general there were no formal reports about differences in gender, the study found that only women farmers lost access to land (*n* = 2), and five out of six farmers who had to halt production completely were women (two women farmers voluntarily stopped work to stay at home to care for family). In both articles, results suggest that women are losing their jobs more readily than men. Future research should examine the reasons and how jobs are lost i.e., whether women are leaving of their own free will or whether they are laid off in greater proportion than men.

Most predictive articles reported likelihoods of higher inequalities to be experienced by women related to COVID-19 and food security (*n* = 10/15). Many predicted that more women would experience increased food insecurity under COVID-19 than men [9,34,36,38], many also warned of increases in gender-based violence [14,18,30,32,37]. However, Tom and Chipenda [30] did not offer actual evidence of such occurrences and a few documents seemed to base their predictions on global statistics [17,32,37], which did not necessarily provide an actual picture of the current conditions in Africa. Breakfast’s and Nomarwayi’s predictions of increased violence linked to food security and the pandemic suggested that this was more likely to occur in rural settings, where police presence was typically lower, indicating a need to target future research in such settings [14]. Overall, the number of predictive documents that reported increases in female and male inequalities underlined the urgent need for research focusing on on-the-ground, real-time evidence collection in African nations, particularly in rural regions. Understanding how women deal with food insecurity, pandemics, and power struggles can also define how these factors impact their health and capacity to continue providing, especially for their children.

#### 3.2.3. Lower or Unknown Reporting

Only a single evidence study and none of the predictive studies alluded to a possible lower rate of inequality experienced by women due to the COVID-19 pandemic and food security-related conditions. Surveys conducted by Kansiime et al. found that both Kenya and Uganda food insecurity increased (especially in low-income households), but that the only clear difference between women and men was that men reported COVID-attributed income loss 11% more than female respondents [23]. However, surveys were conducted through the internet, with most respondents having tertiary education levels (85% in Kenya and 97% in Uganda), and 50% to 73% of respondents were salaried employees. Therefore, a large portion of the vulnerable and rural populations was likely missed. The authors also indicated that two thirds of the respondents were male (from mainly male headed households), which questioned how many individuals and women of the vulnerable or rural population (in this case women) were missed entirely.

The final four documents considered in the study (evidence *n* = 2, predictive *n* = 2) were categorized as drawing unknown conclusions yet were worthy of consideration. Shupler et al. reported that since the onset of the pandemic, nine out of ten households considered themselves to be more food insecure due to loss of income [22]. The main issue with this article was that while women versus men were considered in the initial baseline survey, data were combined in the follow-up survey, making it impossible to determine who was most affected. While Saalim et al. presented a very structured PRISMA media review that focused somewhat on women and pandemic food security specifically, the authors made many predictions based on information gathered from the previous Ebola epidemic [19]. Much of the media reviewed used terms such as “at-risk” or “serious concerns about” when referring to the safety and security of women. Children might have been the most vulnerable in terms of food security and violence (assumingly, both girls and boys were reported together). They only mentioned one article that reported gender-based violence both before and after the onset of the COVID-19 pandemic (i.e., in Liberia). However, upon closer investigation, they did not provide clear details on comparisons between pre- and post-COVID numbers. Their study showed that the media, although mainly anecdotal in nature, reported a great deal of information concerning current gender-based disparities. Caution should be taken when using media materials, as there could be bias. A media-review should be used with a critical eye on the sources of the materials.

Badu et al. did not examine food insecurity-driven gender disparities of the pandemic but mentioned the probability of increased gender-based violence [33]. The authors suggested that mitigation protocols should be put in place to help any potential food security or gender disparity issues. They also mentioned the need to support vulnerable groups, yet they do not specifically include women as being part of vulnerable groups (instead they indicate that they are referring to low income, senior and disabled individuals). Lung’aho also mentioned in passing that food security policy should focus on gender as well as to allow for marginalized people to actively participate in the crafting of such policy without offering up much information on what the status of either is currently [35]. Mitigation strategies being implemented based on predictions are one way to help women during the COVID-19 crisis, but we must also consider where and how such strategies are initiated, and if they are working or even utilized.

#### 3.2.4. Limitations of the Review and Needs

There are several limitations that apply to this study, including the decision to completely disregard media reports. In some cases, media reports may be the only way to access information that applies to some of the more remote areas of Africa, especially during the pandemic. The lack of evidence-based information from remote rural areas and/or vulnerable populations does not mean that these individuals are not experiencing insecurities and inequalities, only that they have not yet been reported on reliability or formally recognized. Saalim et al., for example, used such a media review [19]. They identified women and children as the most discussed vulnerable groups in media during the pandemic and food insecurity and poverty as cross-cutting issues for the vulnerable populations. While their data may suggest self-reporting for occurrences of gender-based violence and other health issues, bias may exist in such analysis and may not explain the root causes of the issues.

This review shows that a clear disparity exists in the number of available statistics in terms of how many studies and reports disseminate clear and consistent gender information. Obtaining data that include separation by sex as well as actual methodological reporting is crucial to better understand the situation on the ground and push for the implementation of gender-sensitive policy changes. Since PRISMA protocols were used to search the English literature, it may also be beneficial to conduct searches for French language publications, including more local journals, given that many West African countries are mainly French speaking. During any pandemic or other disasters, there is a tendency to rapidly acquire data to demonstrate impacts without having a detailed understanding of the conditions on the ground. Unfortunately, most measures, policies and even aid are based on such rapid assessments that do not consider gender issues, power relations between men and women, and social classes, among many aspects of a crisis such as a pandemic.

The COVID-19 pandemic in Africa has exposed the need to better understand what is happening on-the-ground in terms of the food insecurity and other inequalities faced by women. There is a need to explore not just how we think individuals may be affected but how they are being effectively affected, and how to create better reporting options and support for occurrences of gender-based violence and the like. There is an urgent need to better understand the needs of individuals, particularly women (e.g., farmers, caregivers, traders) and to apply gender specific variables/questions to all research that seeks to address socially negative, pandemic-related effects on their health, wellbeing, and conditions due to food insecurity. When investigating concepts such as food security, there is an additional need to address all four pillars (i.e., availability, access, utilization, and stability), as each pillar is intimately connected to the other.

## 4. Conclusions

This study investigates what is currently known about the association between the COVID-19 pandemic in terms of the restrictive measures and the effects on food security, and gender disparities as they exist in Africa. Results demonstrated that disparities exist in the reporting of data regarding rural versus urban and women versus men. It was further found that a large portion of the literature focuses on predictions surrounding what may happen during the COVID-19 pandemic, most of which predicted women would be subjected to increased inequality. No literature was found about the impact of the pandemic-related stress on power relationships between genders. Less literature investigated actual evidence that supported what was being predicted; however, reporting disparities between gender within communities and households and a general lack of on-the-ground, well-designed, evidence-based research suggests that we are missing the full picture. The study also highlighted a lack of evidence and studies conducted in West Africa, and furthermore an absence of studies on gender impact during COVID-19 in Burkina Faso and Senegal, especially on women, their food insecurity, and their health. Further studies that focus on collecting disaggregated data from rural settings that also consider media reports prior to designing research questions is recommended.

## Figures and Tables

**Table 1:** A summary of the extraction information for the 25 retained articles including category, location, type, pillars addressed, direction of women inequality changes, and article citations.

Category	Location	Type ^1^	Pillar ^2^				Inequalities ^3^	Citations
			Availability	Access	Utilization	Stability		
Evidence	Ghana	QnPR	x	x	x	x	Neutral	[21]
	Kenya	QnPR	x	x	x	x	Unknown	[22]
	Kenya, Uganda	QnPR	x	x			Lower	[23]
	Nigeria	QnGR	x	x	x	x	Neutral	[24]
		QnPR		x			Mixed	[25]
		QnPR		x			Neutral	[26]
		QnPR		x			Higher	[27]
	South Africa	QnGR		x			Neutral	[28]
		QnGR	x			x	Higher	[29]
	West Africa	QlPRe	x	x	x	x	Unknown	[19]
Predictive	Nigeria	QlPRe	x	x	x	x	Higher	[18]
	Zimbabwe	QlPO		x			Higher	[30]
	South Africa	QlPRe	x	x	x	x	Higher	[14]
		QnPR		x			Neutral	[31]
		QlGO	x	x		x	Higher	[17]
	South/East Africa	QnGRp	x	x	x	x	Mixed	[15]
	Sub-Saharan Africa	QnPR	x	x	x	x	Neutral	[16]
		QlGRe	x	x	x	x	Higher	[32]
	Africa General	QlGRe		x			Unknown	[33]
		QlPR			x		Higher	[9]
		QlPO		x	x		Higher	[34]
		QlPO	x	x	x	x	Unknown	[35]
		QlGO	x	x		x	Higher	[36]
		QlPR	x	x	x	x	Higher	[37]
		QlRp	x	x	x	x	Higher	[38]

^1^ Type: Qn = quantitative, Ql = qualitative, P = peer reviewed, G = grey, R = research, Re = review, Rp = report, O = opinion; ^2^ As it relates to gender; ^3^ inequalities experienced by women as compared to men (neutral = no change, lower = women experienced less inequality than normal, higher = women experienced more inequality than normal, mixed = context-dependent changes between women and men both lower and higher, unknown = insufficient evidence to assign a rating).

## Data Availability

The data presented in this study are openly available in Scholars Portal Dataverse at https://doi.org/10.5683/SP3/FJXROG (accessed on 13 January 2022).

## Supplementary Materials

The following supporting information can be downloaded at: https://www.mdpi.com/article/10.3390/ijerph19042065/s1, Document S1: PRISMA Flow Diagram.
