# Supplementary material for: Do COVID-19 and Food Insecurity Influence Existing Inequalities between Women and Men in Africa?
Source: Int J Environ Res Public Health. 2022 Feb 12;19(4):2065. doi: 10.3390/ijerph19042065 (PMC8871765; doi:10.3390/ijerph19042065)
Supplement: Supplementary file 1 [file ijerph-19-02065-s001.zip › ijerph-1577276-supplementary.pdf]

Supplementary File S1

Rapid review PRISMA flowchart results for: How do Covid-19 and food insecurity influence existing inequalities between women and men in Africa? (search ‘a’ was conducted in March 2021, search ‘b’ in October 2021).

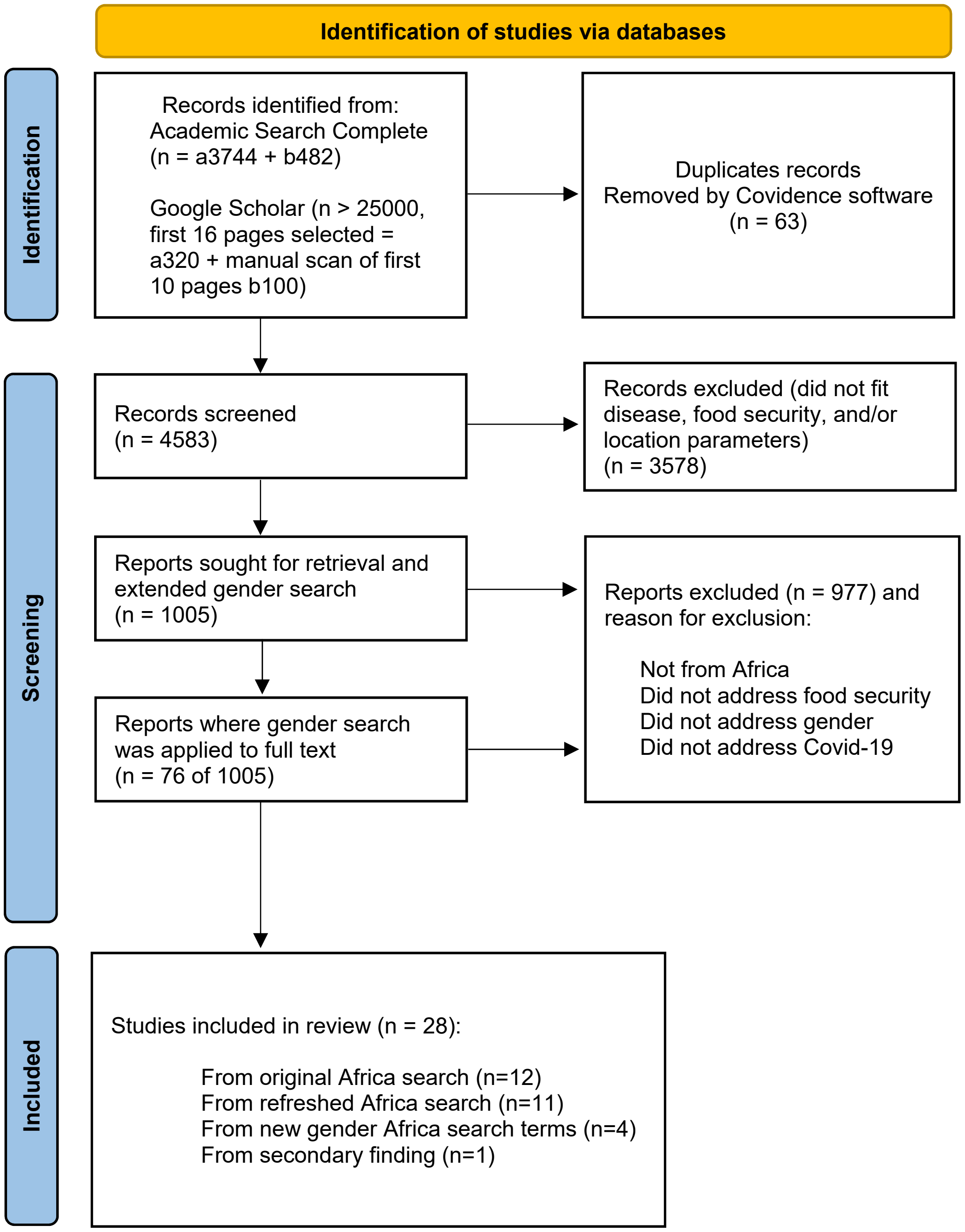

Adapted From: Page MJ, McKenzie JE, Bossuyt PM, Boutron I, Hoffmann TC, Mulrow CD, et al. The PRISMA 2020 statement: an updated guideline for reporting systematic reviews. BMJ 2021;372:n71. doi: 10.1136/bmj.n71.
